# Visual Evaluation of Image Quality of a Low Dose 2D/3D Slot Scanner Imaging System Compared to Two Conventional Digital Radiography X-ray Imaging Systems

**DOI:** 10.3390/diagnostics11101932

**Published:** 2021-10-19

**Authors:** Ahmed Jibril Abdi, Bo Mussmann, Alistair Mackenzie, Oke Gerke, Gitte Maria Jørgensen, Thor Eriksen Bechsgaard, Janni Jensen, Lone Brunshøj Olsen, Poul Erik Andersen

**Affiliations:** 1 Department of Clinical Research, University of Southern Denmark, 5000 Odense, Denmark; bo.mussmann@rsyd.dk (B.M.); oke.gerke@rsyd.dk (O.G.); janni.jensen@rsyd.dk (J.J.); peandersen@health.sdu.dk (P.E.A.); 2 Department of Clinical Engineering, Region of Southern Denmark, 5000 Odense, Denmark; 3 Department of Radiology, Odense University Hospital, 5000 Odense, Denmark; gitte.maria.jorgensen@rsyd.dk (G.M.J.); thor.eriksen.bechsgaard@rsyd.dk (T.E.B.); lone.brunshoej.christiansen@rsyd.dk (L.B.O.); 4 National Coordinating Centre for the Physics of Mammography, Royal Surrey NHS Foundation Trust, Guildford GU2 7XX, UK; alistairmackenzie@nhs.net; 5 Department of Nuclear Medicine, Odense University Hospital, 5000 Odense, Denmark

**Keywords:** visual grading analysis, slot scanner imaging system, DR imaging systems, image quality assessment, chest examination, knee examination

## Abstract

The purpose of this study was to assess the image quality of the low dose 2D/3D slot scanner (LDSS) imaging system compared to conventional digital radiography (DR) imaging systems. Visual image quality was assessed using the visual grading analysis (VGA) method. This method is a subjective approach that uses a human observer to evaluate and optimise radiographic images for different imaging technologies. Methods and materials: ten posterior-anterior (PA) and ten lateral (LAT) images of a chest anthropomorphic phantoms and a knee phantom were acquired by an LDSS imaging system and two conventional DR imaging systems. The images were shown in random order to three (chest) radiologists and three experienced (knee) radiographers, who scored the images against a number of criteria. Inter- and intraobserver agreement was assessed using Fleiss’ kappa and weighted kappa. Results: the statistical comparison of the agreement between the observers showed good interobserver agreement, with Fleiss’ kappa coefficients of 0.27–0.63 and 0.23–0.45 for the chest and knee protocols, respectively. Comparison of intraobserver agreement also showed good agreement with weighted kappa coefficients of 0.27–0.63 and 0.23–0.45 for the chest and knee protocols, respectively. The LDSS imaging system achieved significantly higher VGA image quality compared to the DR imaging systems in the AP and LAT chest protocols (*p* < 0.001). However, the LDSS imaging system achieved lower image quality than one DR system (*p* ≤ 0.016) and equivalent image quality to the other DR systems (*p* ≤ 0.27) in the knee protocol. The LDSS imaging system achieved effective dose savings of 33–52% for the chest protocol and 30–35% for the knee protocol compared with DR systems. Conclusions: this work has shown that the LDSS imaging system has the potential to acquire chest and knee images at diagnostic quality and at a lower effective dose than DR systems.

## 1. Introduction

The low-dose 2/3D slot scanner (LDSS) imaging system can perform full digital skeletal radiography and expose patients to a very low dose of radiation. The two-dimensional (2D) images of the LDSS imaging system make it possible to acquire three-dimensional (3D) model images that can be used for angulation and distance measurements [1]. The LDSS imaging system image detector operates at a considerably higher sensitivity and more effectively reduces scattered radiation compared to conventional DR imaging systems [2,3]. However, the LDSS imaging system is not commonly used for general radiology examinations. The system is mainly used to obtain overview images in patients with scoliosis, for leg length measurements and orthopaedic practices, such as leg length discrepancy, sagittal balance, and scoliosis [4,5,6,7,8]. The present study further elaborates on previous studies that evaluated the image quality of the LDSS imaging system using different image quality assessment techniques and compared it the image quality of conventional DR imaging systems. One of these studies evaluated the contrast detail resolution and patient dose savings of the LDSS imaging system compared to conventional DR imaging systems [9]. The study found that the LDSS imaging system exposed less radiation to patients with sufficient image quality compared to conventional DR imaging systems. Test objects were used in the previous study [9] as a simple measure of image quality. In another previous study, quantitative image quality was assessed using the same systems and the same clinical protocols and revealed that the LDSS imaging system had better quantitative image quality than the DR systems for chest and knee protocols [10]. However, there is still a need for more image quality assessments using more clinically relevant phantoms.

In this study, the X-ray images of the imaging systems were obtained using two clinical examination protocols: standing knee and chest examination protocols. Chest radiography is one of the most commonly accessible radiological examinations and accounts for about 20% of all radiological examinations [11]. The knee X-ray is also an essential and commonly used musculoskeletal examination for diagnosing knee pathology [12].

Observer image quality assessment and performance studies play an essential role in optimising imaging systems to produce images with higher diagnostic accuracy [13]. The quantification of image quality depends on the observers’ perception of the quality of the X-ray images as well as on the observer’s experience [14]. Several methods for quantifying image quality exist, and of which have their advantages and disadvantages. Visual grading of the clarity of key anatomical or pathological structures is a method characterised by visual appearance and important discriminating characteristics. Visual grading also helps to quantify subjective views and enable their analysis.

Visual grading analysis (VGA) is an important and useful observer image quality evaluation method for evaluating the image quality and diagnostic performance of X-ray imaging systems [15,16]. VGA assesses the image quality of imaging systems and can be performed using two main scoring methods: relative gradation and absolute gradation [17]. In the relative VGA method, the observers score the quality of radiographic images by comparing one or more reference images to assess whether the quality of the image is better or worse than the reference images. In the absolute VGA method, the observers score the image quality of radiographic images without using reference images [18] and, instead, score a set of clinically relevant descriptors of the anatomy shown in the images. 

In this study, we used the absolute VGA scoring method to evaluate the image quality of thoracic and knee phantoms in LDSS and two DR imaging systems. The VGA image quality assessment method is commonly used in medical imaging technologies and clinical protocols to evaluate the diagnostic capability of the technology and the quality of the images [19]. The VGA method has several advantages, including a detailed image quality assessment and its low-cost [20]. Several previous studies have shown that the VGA method is suitable for evaluating the image quality of chest examinations [14,21,22]. Moreover, the VGA method has been used to assess image quality in radiographic examinations of the extremities, including knee protocols [23,24]. 

In addition to evaluating the image quality of these systems, we calculated the effective dose (ED) to patients for all imaging systems in both chest and knee protocols. The calculated ED for the patient from LDSS imaging systems was compared with the ED from DR imaging systems in both chest and knee protocols. In a previous study, the ED for patients was calculated for LDSS imaging system and DR systems in both chest and knee protocols using Polymethyl Methacrylate (PMMA) patient-equivalent phantoms [9]. The ED was calculated in the present study using the clinical human-like phantoms that were also used to evaluate VGA image quality.

The present study aims to assess the quality of anthropomorphic chest phantom and knee phantom images of the LDSS imaging system compared to conventional DR imaging systems using the VGA image quality evaluation method.

## 2. Materials and Methods

An anthropomorphic chest phantom (LungMan, Kyoto Kagaku Co., Ltd., Kyoto, Japan) with an additional associated fat layer was used to evaluate the chest X-ray examination of soft tissue structures. The phantom simulates clinical thorax of average patient size for the chest examination protocol. 

**Figure 1:**
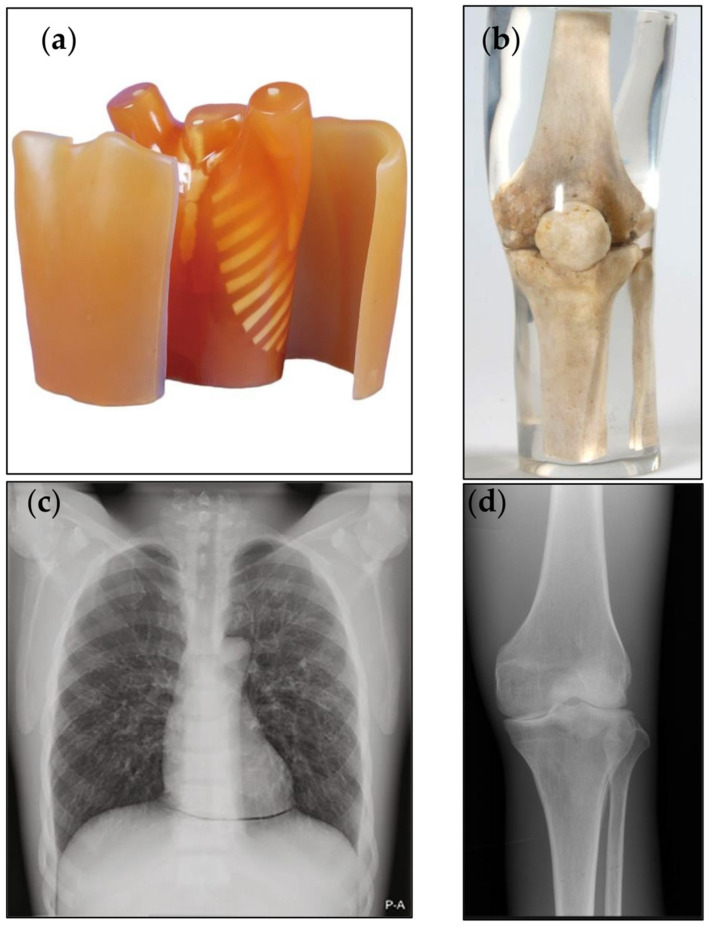
The frontal view of (**a**) photographic image of the chest anthropomorphic phantom (**b**) photographic image of the knee phantom (**c**) radiographic image of the chest anthropomorphic phantom and (**d**) radiographic images of the knee phantom.

A knee phantom that incorporates native bones was used to assess the quality of images of the bone structures. The photographic images and radiographic images of the phantoms are shown in the Figure 1.

The imaging systems used to assess the clinical phantom images are listed below. These systems were also used in a previous study evaluating the contrast detail resolution of these systems [9].

LDSS: the LDSS imaging system (EOS SA, Paris, France) (www.eos-imaging.com (accessed on 1 October 2021)) allows the acquisition of two simultaneous X-ray images: the frontal (posterior-anterior) and lateral projections. LDSS imaging system incorporates a higher sensitivity gaseous detector with a pixel size of 254 µm.

DR system 1: Philips DigitalDiagnost (DiDi) DR X-ray imaging system (Philips Healthcare, Best, the Netherlands), with a Trixell flat panel wall stand and a Caesium Iodide (CsI) detector, Pixel size 143 µm). 

DR system 2: Siemens Ysio DR X-ray imaging system (Siemens Healthineers GmbH, Forchheim, Germany) with a Trixell flat panel wall stand and a CsI detector, with a pixel size of 139 µm. 

A Piranha 657 solid-state dosimeter (RTI Group, Mölndal, Sweden) was used to measure the patient’s entrance exposure and verify the system’s dose area product (DAP) meters. Routine annual quality control was performed on the DAP meter of the system.

Viewer for Digital Evaluation of X-ray images (ViewDEX) version 2.48 image quality scoring software was used to score and assess the image quality of all imaging systems and clinical examination configurations [25,26]. ViewDEX is a Java-based software used for presenting and evaluating medical images in observer performance studies [27]. ViewDEX software is also used for the analysis and scoring of radiological image quality. The desired scoring rate and scoring criteria can be set up by reprogramming the files that are included in this software [26,28]. The images can be scored in either a single image or series of images (stacks). 

PCXMC Monte Carlo simulation software version 2.0.1 (the Finnish Radiation and Nuclear Safety Authority, STUK; Helsinki, Finland) was used to calculate the effective dose to patients. The PCXMC Monte Carlo program is currently one of the most suitable simulation software for estimating organ doses in medical imaging [29].

### 2.1. Clinical Examination Protocols and Technical Settings

The clinical protocol setup for all imaging systems is based on a previously conducted optimisation study [9]. The clinical examination settings for all systems used in the present study are listed in Table 1. The default parameters and geometrical settings (exposure parameters) of these three imaging systems, including source image distance (SID), tube voltage (kV), additional filtering, and other relevant parameters in the chest and knee protocols, are summarised in Table 2. The chest images were acquired with the clinically used anti-scatter grid for both DR systems, whereas LDSS images were acquired without anti-scatter grid. The images of the knee phantom were acquired without an anti-scatter grid for all of the imaging systems.

The scan speed of the LDSS imaging system was optimised. The scan speed of the chest protocol was increased from the default (speed 4) to speed 6, and in knee protocol from speed 6 to speed 8 to increase the dose level and achieve higher image quality comparable to the DR imaging systems. 

### 2.2. Image Quality Assessment

Ten radiographic images of the anthropomorphic (chest) and extremity (knee) phantom were acquired in each PA and lateral view for each imaging system. 

Three experienced radiologists practising thoracic radiology and specialising in the reporting of thoracic images scored the 60 anthropomorphic chest phantom images acquired on all three imaging systems in both PA and LAT projections. Similarly, 60 knee images from all three imaging systems were scored by two experienced diagnostic radiographers with postgraduate degrees in appendicular skeletal reporting and a research radiographer. 

The observers scored the chest and knee images using a predefined scoring scale (Table 3) and image quality criteria (Table 4 and Table 5).

To determine intraobserver agreement, two observers repeated the scoring of all images for the knee and chest protocols.

The phantom images of the imaging systems in both chest and knee protocols were presented to the observers in a randomised order. The images were displayed without annotations or DICOM tags during the scoring. Images were scored using a diagnostic monitor with a minimum resolution of 3 megapixels. The VGA grading was undertaken at the same location using the same diagnostic monitor and the same physical circumstances.

Evaluation of the quality of the phantom images was obtained using absolute VGA as a measure of subjective image quality [30,31]. The results of the VGA analysis can be compiled in a score by applying the following equation [17,31]:

(1) $$\mathrm{VGAS}=\frac{\sum_{0,I}S_{c}}{N_{i}N_{o}}$$

where S_c_ = the given individual scores for observer (O) and image (I), N_i_ = total number of images, and N_o_ = total number of observers. 

Absolute VGA in the chest and knee protocols was scored using a five-point scale ranging from poor to excellent image quality (Table 3) [14,17,19,30,32,33].

Technical image quality criteria for the chest acquisition in both PA and LAT projections are introduced in Table 4 [32,34]. All radiologists who score the chest images have approved these criteria.

The image quality criteria for the knee protocols for PA and LAT projections are listed in Table 5. These criteria are based on the European Commission. European guidelines on quality criteria for diagnostic radiographic images and criteria developed in a previous study and were adjusted for the knee protocols [32,35,36]. These criteria have been approved by the reporting radiographers and research radiographer who scored the knee images.

### 2.3. Estimation of the Radiation Exposure to the Patients

The solid-state dosimeter without backscatter was placed in the centre of the entrance of the anthropomorphic patient-equivalent phantom and the knee phantom to measure the incident air kerma (AK) of the patient. For the chest acquisition, the dosimeter is located outside the sensor areas of the detector to avoid interfering with the automatic exposure control (AEC) of the DR imaging systems. However, the chest acquisitions of the LDSS and the knee acquisitions of all imaging systems were performed without AEC mode techniques.

To verify the DAP meters of the imaging systems, the DAP was calculated using product of the irradiated area and the measured AK [9]. The ED was determined using the verified DAP values of the systems according to the International Commission’s approach as outlined in Radiological Protection publication 103 (ICRP, 2007) [37]. ED calculation of the PCXMC software in the LDSS imaging system was obtained using the method described by these previously conducted studies [9,38,39].

### 2.4. Statistical Analysis

Descriptive statistics for categorical variables were generated using frequencies and respective percentages. The intraobserver agreement of scoring was assessed using weighted kappa statistics, which is the agreement among repeated administrations of a scoring performed by a single observer [40,41]. The interobserver agreement for the multiple observers for each criterion was assessed using Fleiss’ kappa statistics to obtain agreement that yields a score of homogeneity or consensus across the images quality criteria [42,43,44,45,46,47]. To compare the scored VGA values across the systems for each criterion, two-way analysis of variance (ANOVA) was used [48,49]. The level of statistical significance was 5%. All analyses were performed using the Statistical Package for the Social Sciences (SPSS), Release 26.0.0.0, New York, NY, USA.

## 3. Results

Fleiss’ kappa interobserver agreement of chest PA/LAT protocols for each image quality criteria is shown in Table 6.

Fleiss’ Kappa interobserver agreement in the chest LAT protocols for all imaging systems for image quality criteria ranged between 0.27 and 0.55, whereas Fleiss’ kappa interobserver agreement for chest PA protocol ranged between 0.19 and 0.78. These Fleiss kappa interobserver agreement assessments for image quality criteria in both chest PA and LAT protocols varied between slight and substantial [50]. The corresponding Fleiss’ kappa interobserver agreement for each criterion in knee PA/LAT protocol is shown in Table 7. 

Fleiss’ Kappa evaluating interobserver agreement for each criterion and all imaging systems in the knee LAT protocol ranged from 0.23 to 0.45 (fair to moderate agreement). However, Fleiss’ kappa for the criteria in the knee PA ranged from 0.17 to 0.26 (slight to fair agreement). The weighted kappa of the intraobserver agreement for the image quality criteria in chest PA/LAT projections is shown in Table 8.

The intraobserver agreement obtained in the chest protocol was good. The weighted kappa coefficients for intraobserver agreement in the chest LAT projection ranged from 0.13 to 0.31, which can be interpreted as slight to fair agreement. In the chest PA projection, weighted kappa coefficients for the intraobserver agreement ranged from 0.25 to 0.68 (fair to substantial agreement). 

The corresponding weighted kappa for intraobserver agreement for the knee PA/LAT protocol for all imaging systems and VGA image quality criteria are shown in Table 9.

The weighted kappa for agreement between observers obtained in the knee protocol was also good. The intraobserver weighted kappa coefficient of the knee LAT ranged from 0.19 to 0.38 (slight to fair agreement). The intraobserver weighted kappa coefficient for the knee PA projection ranged from 0.13 to 1.00, which can be interpreted as slight to excellent agreement.

### VGA Comparison across the Systems and Image Quality Criteria

VGA scored image quality was compared between imaging systems and image quality criteria using two-way ANOVA. The VGA mean values across the systems and image quality criteria for the chest PA/LAT are presented in Table 10. 

The LDSS imaging system achieved higher mean VGA scores than the DR imaging systems, which were 4.33 and 4.73 for the chest PA and LAT, respectively. DR system 2 achieved the lowest VGA mean values, which were 3.69 and 3.40 for the chest PA and LAT projections, respectively.

Pairwise comparison of the scored VGA means across the systems in chest PA/LAT is shown in the Table 11.

The LDSS imaging system obtained significantly higher VGA mean scores than the DR imaging systems in the chest protocol for both projections (*p* < 0.001). The mean difference between the LDSS imaging system and DR systems in the chest LAT protocol was 0.45 and 0.94 for DR system 1 and DR system 2, respectively. In the chest PA projection, the LDSS imaging system achieved significantly higher VGA mean values than the DR imaging systems. The differences in VGA mean between the LDSS imaging system and the DR imaging systems in the chest PA were 0.833 and 1.05 for DR system 1 and DR system 2, respectively (*p* < 0.001 in both cases).

DR system 1 had a higher VGA score than DR system 2, with VGA mean differences of 0.213 and 0.46 in chest PA and LAT projections, respectively, (*p* = 0.006 in both cases). Thus, DR system 2 achieved the lowest VGA score in the chest protocol.

**Figure 2:**
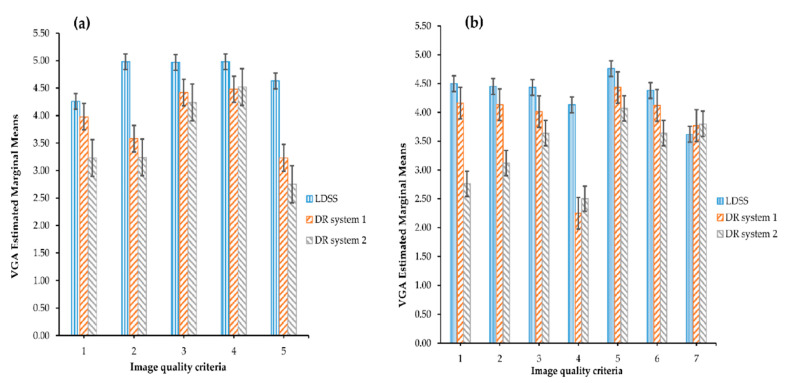
VGA estimated marginal mean comparison across the imaging systems and image quality criteria for (**a**) the chest PA projection and (**b**) the chest LAT projection.

Estimated marginal means of scored VGA for each criterion and all imaging systems in chest PA and LAT protocol are in shown in Figure 2. The standard error bars present the mean error for all values, determining how far the sample mean is likely to be from the population mean.

As shown in Figure 2, the LDSS imaging system scored better mean VGA than the DR systems for all image quality criteria for the chest PA and LAT projections, except for image quality criterion 7 of the chest LAT projection, for which LDSS achieved slightly lower mean VGA than the DR systems.

All observers assigned the best mean VGA scores for the DR imaging systems on image quality criterion 4 in the chest PA projection and on criterion 5 in the chest LAT projection. However, the DR systems achieved the lowest mean VGA scores for image quality criterion 5 in the chest PA projection and for criterion 4 in the chest LAT projection.

The calculated mean values of the scored VGA for knee examination protocol are shown in Table 12.

For the knee PA projection, the DR system achieved the highest mean VGA. The LDSS imaging system achieved the next highest mean VGA, and DR system 2 achieved the lowest mean VGA. DR system 1 achieved the lowest mean VGA for knee LAT projection than LDSS imaging system and DR system 2.

The VGA pairwise comparison of the systems for the knee PA/LAT protocol projections is shown in Table 13. 

The comparison of mean VGA between the imaging systems presented in Table 13 is based on estimated marginal means.

For the knee PA projection, DR system 1 had a better mean VGA than the LDSS imaging system. However, there was no significant difference between the mean VGA of the LDSS imaging system (*p* ≤ 0.27) and DR system 2 (*p* ≤ 0.12) in knee PA projection.

In the knee LAT projection, the LDSS imaging system achieved better mean VGA than the DR system 1. There was no significant difference between the mean VGA of LDSS and DR system 2 in the knee LAT projection (*p* ≤ 0.49). DR system 2 achieved a higher mean VGA than DR system 1 in the knee LAT projection.

**Figure 3:**
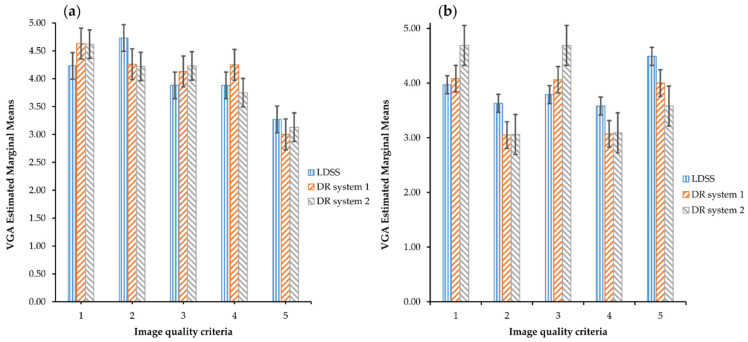
VGA estimated marginal mean comparison across the imaging systems and image quality criteria, (**a**) for the knee PA protocol and (**b**) knee LAT protocol.

The bar chart representation of the estimated marginal means of the assessed VGA for each image quality criteria across the imaging systems in the knee PA/LAT projections is shown in Figure 3.

For knee PA projection, all observers scored lower mean VGA on image quality criterion 5 for all imaging systems. For knee LAT projection, observers scored lower mean VGA on image quality criteria 2 and 4 than the other three image quality criteria for all imaging systems. The mean VGA for criterion 2 of the systems was 3.67, 3.03 and 3.07 for LDSS, DR system 1 and DR system 2, respectively. However, the LDSS imaging system scored higher mean VGA than the DR imaging system on image quality criteria 2 and 4 for the knee PA and image quality criteria 2, 4, and 5 for the LAT projection. In the knee PA projection, the observers have rated lower mean VGA in the image quality criterion 5 than the other criteria in all three imaging systems.

The calculated ED, the measured entrance surface dose to patients, and the ED difference between the LDSS imaging system and the DR imaging systems in chest and knee protocols for all imaging systems are shown in the Table 14.

The calculated ED results for the different imaging systems show that in the chest protocol the LDSS imaging system exposes patients to 52.4% lower ED than DR system 1 and 33.3% less than DR system 2. In the knee protocol, the LDSS imaging system exposes patients to 30.2% and 35.5% lower ED than DR system 1 and 2, respectively.

## 4. Discussion

Fleiss’ kappa and weighted kappa analysis for inter- and intraobserver agreement for all VGA image quality criteria showed good agreement for both chest and knee protocols. Fleiss’ kappa coefficients range from 0.27 to 0.78 and 0.17 to 0.26 for the chest and knee protocols, respectively, while weighted kappa coefficients range from 0.13 to 0.63 and 0.19 to 1.00 for the chest and knee protocols, respectively. This high degree of inter and intraobserver agreement may be partly due to the use of experienced observers from the same institution. All observers gave lower VGA image quality scores on image quality criteria 2 and 4 for the knee LAT projection, especially for the DR systems. Observers also assigned a lower mean VGA image quality score for image quality criterion 5 in the knee PA projection. This could be because the phantom does not produce structures that sufficiently resemble normal patient knee structures.

The VGA image quality results obtained in the chest protocol were relatively higher than the VGA image quality scores in the knee protocol. The mean VGA scores in the chest protocol were in the range of 3.40 to 4.73 on a scale of 1 to 5 for all imaging systems for all quality criteria. However, the overall mean VGA values obtained for the knee protocol were lower than the chest protocol, ranging from 3.72 to 4.07 on a scale of 1 to 5.

In this study, the VGA image quality assessment of image quality depended on the subjectivity of the individual observer, and thus the results were based on the personal evaluation of image quality. However, according to the agreement obtained between observers, there is not much variation between observer agreements in VGA scoring for all image quality criteria.

The main limitation of this study is that although the chest anthropomorphic and knee phantoms are realistic, the resultant images do not perfectly mimic clinical images. Therefore, the observers may miss some structural details when scoring the images. However, phantom images have the advantage of providing observers with uniform and stable phantom images to evaluate without variation in patient size or patient motion. 

The scan time of the LDSS imaging system is longer than the standard exposure time of DR imaging systems for both the chest and knee protocols. This longer scan time to perform a thoracic acquisition on a real patient may result in internal organ motion and patient motion artefacts and depends on the patients’ ability to hold their breath and to remain still for long periods of time. 

No previous studies were found in which the image quality of the LDSS imaging system was subjectively assessed using either patients or phantoms at diagnostic image quality levels. However, the VGA image quality assessment results obtained in the present study are more or less comparable to a previous objective image quality evaluation study using the same imaging systems and clinical protocols [9]. In this previously conducted study, the results of the objective image quality assessment showed that the LDSS imaging system achieved comparable image quality to the DR imaging systems for both chest and knee protocols.

The overall results of the achieved VGA image quality in the present study show that the LDSS imaging system achieved better VGA image quality than the DR imaging systems in both the chest PA and the LAT projection acquisition. Regarding the knee acquisitions, the VGA image quality results also showed that the LDSS imaging system achieved either better or equivalent VGA image quality compared to the DR imaging systems, with the exception that DR system 1 obtained better VGA image quality than the LDSS imaging system for the knee LAT projection. Some differences in VGA image quality were also noted between the DR imaging systems for both chest and knee acquisitions. Based on these overall VGA image quality results, it is suggested that LDSS has the potential to produce radiographs with sufficient diagnostic information in both thoracic and extremity protocols.

As expected, the LDSS imaging system exposed patients to lower ED than the imaging systems from DR for both the chest and knee protocols. The estimated ED values obtained in the current study are similar to the ED values obtained in the previous study [9]. The measured DAP values and the calculated ED obtained in the current study for the knee protocol were slightly lower than in the previous study for all systems. This is because the irradiated field for the PMMA phantom used in the previous study was slightly larger than the irradiated field for the knee phantom.

## 5. Conclusions

This work has shown the potential for the LDSS imaging system to obtain chest and knee images suitable for the acquisition of chest and knee images in a diagnostic radiographic unit. Using experienced radiologists and reporting and research radiographers, we have shown that the images of the LDSS imaging system are generally better than or of the same quality as two DR systems in clinical use, but with the advantage of entailing lower effective dose.

## Figures and Tables

**Table 1:** The list of study protocols and radiation dose level settings used in both patient dose and image quality comparisons of the LDSS and the DR systems.

Systems	Projections	Dose Level	Protocol
DR system 1	PA/LAT	Standard (default)	Chest (thorax)
DR system 2	PA/LAT	Standard (default)	
LDSS	PA/LAT	Medium dose (speed 6 sec.)	
DR system 1	PA/LAT	Standard (default)	Extremity (knee)
DR system 2	PA/LAT	Standard (default)	
LDSS	PA/LAT	High dose (Speed 8 sec.)	

**Table 2:** Exposure settings in chest and knee examinations for all imaging systems.

Imaging Systems	Tube Voltage [kV]	SID (cm)	Tube Current (mA)	Tube Load (mAs)	Exposure Mode	AF Al/Cu (mm)	Examination Protocols
LDSS speed 6	90	130	280	n/a	Manual	0/0.1	Chest
DR system 1	133	250	n/a	1.6	AEC	1/0.2	
DR system 2	145	300	n/a	1.8	AEC	0/0.2	
LDSS speed 8	68	130	400	n/a	Manual	0/0	Knee
DR system 1	57	110	n/a	8.5	Manual	0/0	
DR system 2	63	115	n/a	6.3	Manual	0/0	

n/a = not applicable, AEC = automatic exposure control, SID = source to image distance, AF = additional filtration, Al = aluminium filtration and Cu = copper.

**Table 3:** VGA-based scale for scoring image quality.

Scale	Image Scoring Scale	Description
1	Poor image quality:	Image not usable, loss of information
2	Restricted image quality:	Relevant limitations for clinical use, clear loss of information
3	Sufficient image quality:	Moderate limitations for clinical use, no substantial loss of information
4	Good image quality:	Minimal limitations for clinical use
5	Excellent image quality:	No limitations for clinical use

**Table 4:** Image quality criteria for the chest examination protocols in both PA and LAT projections.

Criteria	Technical Image Quality Criteria		Projections
1	Visualisation of:	Vascular pattern of lungs and peripheral vessels	PA
2		Trachea and proximal bronchi	
3		Borders of the heart and the aorta	
4		Diaphragm and lateral costophrenic angles	
5		Spine through the heart shadow	
1	Visualisation of:	Thoracic spine	LAT
2		Trachea	
3		Costophrenic angles	
4		Sternum	
5		Diaphragm	
6		Mediastinum	
7		Posterior border of the heart and aorta	

**Table 5:** Image quality criteria for the knee examination protocol in both PA and LAT projections.

Criteria	Image Quality Criteria Definitions	Technical Image Quality	Projection
1	Homogeneity in soft tissue, lateral to the femoral condyle	Noise	knee PA
2	Sharpness of trabeculae in the medial femoral condyle	Spatial resolution	
3	Sharpness of the demarcation between substantia spongiosa and substantia compacta in the femur above patella	Contrast small structure	
4	Visualization of patella through the femur	Low-contrast resolution and noise	
5	Visualization of the lateral intra-articular femoral condyle	Contrast large structure	
1	Homogeneity in soft tissue posterior to the knee joint	Noise	knee LAT
2	Visualization of head of fibulae behind the tibia	Low-contrast resolution and noise	
3	Visualization of patella	Contrast large structure	
4	Sharpness of trabeculae in the tibial metaphysis	Spatial resolution	
5	Sharpness of the demarcation between substantia spongiosa and substantia compacta in the anterior femur above patella	Contrast small structure	

**Table 6:** Fleiss’ kappa assessing interobserver agreement with an asymptotic 95% confidence interval (CI) for each criterion in chest PA/LAT protocols.

Criteria	1	2	3	4	5	6	7	Projections
**Kappa**	**0.60**	**0.68**	**0.73**	**0.19**	**0.78**	n/a		chest PA
**95% CI**	0.39–0.80	0.51–0.85	0.52–0.93	0.03–0.35	0.61–0.93			
***p*** **-value**	<0.001	<0.001	<0.001	0.02	<0.001			
**Kappa**	**0.55**	**0.36**	**0.56**	**0.47**	**0.44**	**0.63**	**0.27**	chest LAT
**95% CI**	0.49–0.61	0.19–0.52	0.41–0.72	0.30–0.63	0.26–0.61	0.45–81	0.13–0.41	
***p*** **-value**	<0.001	<0.001	<0.001	<0.001	<0.001	<0.001	<0.001	

n/a = not applicable.

**Table 7:** Fleiss’ kappa assessing interobserver agreement with an asymptotic 95% CI for each criterion in knee PA/LAT protocols.

Criteria	1	2	3	4	5	Projections
**Kappa**	**0.45**	**0.29**	**0.31**	**0.39**	**0.23**	knee PA
**95% CI**	0.26–0.64	0.09–0.49	0.13–0.48	0.19–0.59	0.10–0.36	
***p*** **-value**	<0.001	0.004	<0.001	<0.001	<0.001	
**Kappa**	**0.24**	**0.18**	**0.20**	**0.17**	**0.26**	knee LAT
**95% CI**	0.06–0.42	0.03–0.33	0.01–0.33	0.05–0.29	0.07–0.44	
***p*** **-value**	0.009	0.016	0.002	0.007	0.007	

**Table 8:** Weighted kappa assessing intraobserver agreement for each image quality criterion in the chest PA/LAT protocol.

Criteria	1	2	3	4	5	6	7	Projections
**Kappa**	**0.25**	**0.26**	**0.28**	**0.27**	**0.63**	n/a		chest PA
**95% CI**	0.07–0.44	−0.11–0.42	−0.09–0.64	−0.06–0.61	0.49–0.78			
***p*** **-value**	0.02	0.01	<0.001	<0.001	<0.001			
**Kappa**	**0.13**	**0.31**	**0.28**	**0.26**	**0.17**	**0.14**	**0.26**	chest LAT
**95% CI**	−0.22–0.48	0.11–0.51	−0.15–0.70	0.05–0.46	−0.02–0.36	−0.10–0.29	−0.09–0.60	
***p*** **-value**	0.10	<0.001	0.04	0.04	0.05	0.05	0.02	

n/a = not applicable.

**Table 9:** Weighted kappa assessing intraobserver agreement for each image quality criterion in the knee PA/LAT protocol.

Criteria	1	2	3	4	5	Projections
**Kappa**	**0.38**	**0.13**	**0.21**	**0.21**	**1.00**	knee PA
**95% CI**	0.16–0.60	−0.13–0.35	−0.23–0.64	−0.01–0.42	n/a	
***p*** **-value**	<0.001	0.41	0.07	0.04	<0.001	
**Kappa**	**0.27**	**0.19**	**0.28**	**0.21**	**0.34**	knee LAT
**95% CI**	0.01–0.54	−0.05–0.44	−0.12–0.68	−0.10–0.53	−0.07–0.75	
***p*** **-value**	0.03	0.01	<0.001	0.02	0.01	

n/a = not applicable.

**Table 10:** Means of VGA value for all imaging systems for the chest PA/LAT protocol.

Systems	Mean	95% CI	Projections
**LDSS**	4.73	4.64–4.83	chest PA
**DR system 1**	3.90	3.80–3.99	
**DR system 2**	3.69	3.59–3.78	
**LDSS**	4.33	4.24–4.42	chest LAT
**DR system 1**	3.86	3.37–3.95	
**DR system 2**	3.40	3.31–3.49	

**Table 11:** Comparison of the mean VGA across the imaging systems in chest PA and LAT protocol.

System Comparison	Mean Difference	Std. Error	Sig. ^b^	95% CI	Projections
LDSS vs. DR system 1	0.83	0.07	<0.001	0.67–0.99	chest PA
LDSS vs. DR system 2	1.05	0.07	<0.001	0.88–1.20	
DR system 1 vs. DR system 2	0.21	0.07	0.006	0.05–0.38	
LDSS vs. DR system 1	0.47	0.07	<0.001	0.32–0.63	chest LAT
LDSS vs. DR system 2	0.94	0.07	<0.001	0.78–1.09	
DR system 1 vs. DR system 2	0.46	0.07	<0.001	0.31–0.62	

Based on estimated marginal means. ^b^ Adjustment for multiple comparisons: Bonferroni.

**Table 12:** Means of mean VGA values, upper and lower bound of 95% CI for all imaging systems in the knee PA/LAT protocol.

Systems	Mean	95% CI	Projections
**LDSS**	3.86	3.78–3.94	knee PA
**DR system 1**	4.07	3.99–4.14	
**DR system 2**	3.95	3.88–4.03	
**LDSS**	3.85	3.79–3.92	knee LAT
**DR system 1**	3.72	3.65–3.79	
**DR system 2**	3.92	3.85–3.99	

**Table 13:** Comparison of the mean VGA across the imaging systems in knee PA and LAT protocols.

System Comparison	Mean Difference	Std. Error	Sig. ^b^	95% CI	Projections
**LDSS vs. DR system 1**	−0.21	0.069	0.001	−0.34–0.07	knee PA
**LDSS vs. DR system 2**	−0.09	0.069	0.27	−0.23–0.04	
**DR system 1 vs. DR system 2**	0.11	0.069	0.12	−0.02–0.25	
**LDSS vs. DR system 1**	0.13	0.07	0.016	0.02–0.25	knee LAT
**LDSS vs. DR system 2**	−0.07	0.07	0.49	−0.18–0.05	
**DR system 1 vs. DR system 2**	−0.2	0.07	<0.001	−0.31–−0.09	

Based on estimated marginal means. ^b^ Adjustment for multiple comparisons: Bonferroni.

**Table 14:** Calculated patient ED for the chest and knee protocols in all imaging systems, ESD = entrance surface dose and ED differences between the LDSS imaging system and DR imaging systems.

Imaging Systems	ESD (mGy)	DAP (mGycm^2^)	ED (µSv)	ED Difference (%) from LDSS	Protocol	Projections
**LDSS Speed 6**	0.14	142.21	19.76		chest	PA
	0.15	153.22	21.55			LAT
**Total**	**0.29**	**295.42**	**41.31**			
**DR system 1**	0.16	168.93	39.04			PA
	0.19	193.45	47.71	**52.4**		LAT
**Total**	**0.35**	**361.38**	**86.80**			
**DR system 2**	0.15	158.00	21.00			PA
	0.17	179.86	40.90	**33.3**		LAT
**Total**	**0.32**	**337.86**	**61.90**			
**LDSS Speed 8**	0.17	54.91	0.0063		knee	PA
	0.17	55.40	0.0064			LAT
**Total**	**0.34**	**110.31**	**0.0127**			
**DR system 1**	0.18	58.25	0.0099			PA
	0.19	59.33	0.0089	**35.5**		LAT
**Total**	**0.37**	**117.58**	**0.0197**			
**DR system 2**	0.17	56.24	0.0086			PA
	0.18	57.05	0.0096	**30.2**		LAT
**Total**	**0.35**	**113.29**	**0.0182**			

## Data Availability

Results and data of this study were not provided in any public places. The phantom images, calculations, observer scoring and reports were archived in a personal computer hard-drive.
