# Correction: Abdi et al. Visual Evaluation of Image Quality of a Low Dose 2D/3D Slot Scanner Imaging System Compared to Two Conventional Digital Radiography X-ray Imaging Systems. *Diagnostics* 2021, *11*, 1932

**DOI:** 10.3390/diagnostics12051144

**Published:** 2022-05-05

**Authors:** Ahmed Jibril Abdi, Bo Mussmann, Alistair Mackenzie, Oke Gerke, Gitte Maria Jørgensen, Thor Eriksen Bechsgaard, Janni Jensen, Lone Brunshøj Olsen, Poul Erik Andersen

**Affiliations:** 1 Department of Clinical Research, University of Southern Denmark, 5000 Odense, Denmark; bo.mussmann@rsyd.dk (B.M.); oke.gerke@rsyd.dk (O.G.); janni.jensen@rsyd.dk (J.J.); peandersen@health.sdu.dk (P.E.A.); 2 Department of Clinical Engineering, Region of Southern Denmark, 5000 Odense, Denmark; 3 Department of Radiology, Odense University Hospital, 5000 Odense, Denmark; gitte.maria.jorgensen@rsyd.dk (G.M.J.); thor.eriksen.bechsgaard@rsyd.dk (T.E.B.); lone.brunshoej.christiansen@rsyd.dk (L.B.O.); 4 National Coordinating Centre for the Physics of Mammography, Royal Surrey NHS Foundation Trust, Guildford GU2 7XX, UK; alistairmackenzie@nhs.net; 5 Department of Nuclear Medicine, Odense University Hospital, 5000 Odense, Denmark

In the original publication [1], there was a mistake in the Figure 2 on page nine of the paper. Figures 2 and 3 are duplicated, and the corrected version of Figure 2 is missing. This correct figure appears below. The authors apologize for any inconvenience caused and state that the scientific conclusions are unaffected. This correction was approved by the Academic Editor. The original publication has also been updated.

## Figures and Tables

**Figure 2:**
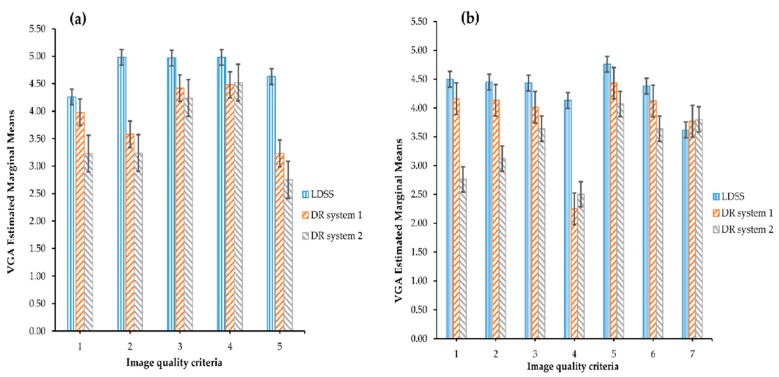
VGA estimated marginal mean comparison across the imaging systems and image quality criteria for (**a**) the chest PA projection and (**b**) the chest LAT projection.
